# Prenatal Air Pollution Exposure and Newborn Blood Pressure

**DOI:** 10.1289/ehp.1307419

**Published:** 2015-01-27

**Authors:** Lenie van Rossem, Sheryl L. Rifas-Shiman, Steven J. Melly, Itai Kloog, Heike Luttmann-Gibson, Antonella Zanobetti, Brent A. Coull, Joel D. Schwartz, Murray A. Mittleman, Emily Oken, Matthew W. Gillman, Petros Koutrakis, Diane R. Gold

**Affiliations:** 1 Department of Environmental Health, Harvard School of Public Health, Boston, Massachusetts, USA; 2 Julius Center for Health Sciences and Primary Care, University Medical Center Utrecht, Utrecht, the Netherlands; 3 Obesity Prevention Program, Department of Population Medicine, Harvard Medical School and Harvard Pilgrim Health Care Institute, Boston, Massachusetts, USA; 4 Department of Geography and Environmental Development, Ben-Gurion University of the Negev, Beer Sheva, Israel; 5 Department of Biostatistics, Harvard School of Public Health, Boston, Massachusetts, USA; 6 Channing Laboratory, Department of Medicine, Brigham and Women’s Hospital and Harvard Medical School, Boston, Massachusetts, USA; 7 Cardiovascular Epidemiology Research Unit, Department of Medicine, Beth Israel Deaconess Medical Center and Harvard Medical School, Boston, Massachusetts, USA; 8 Department of Nutrition, Harvard School of Public Health, Boston, Massachusetts, USA

## Abstract

**Background:**

Air pollution exposure has been associated with increased blood pressure in adults.

**Objective:**

We examined associations of antenatal exposure to ambient air pollution with newborn systolic blood pressure (SBP).

**Methods:**

We studied 1,131 mother–infant pairs in a Boston, Massachusetts, area pre-birth cohort. We calculated average exposures by trimester and during the 2 to 90 days before birth for temporally resolved fine particulate matter (≤ 2.5 μm; PM_2.5_), black carbon (BC), nitrogen oxides, nitrogen dioxide, ozone (O_3_), and carbon monoxide measured at stationary monitoring sites, and for spatiotemporally resolved estimates of PM_2.5_ and BC at the residence level. We measured SBP at a mean age of 30 ± 18 hr with an automated device. We used mixed-effects models to examine associations between air pollutant exposures and SBP, taking into account measurement circumstances; child’s birth weight; mother’s age, race/ethnicity, socioeconomic position, and third-trimester BP; and time trend. Estimates represent differences in SBP associated with an interquartile range (IQR) increase in each pollutant.

**Results:**

Higher mean PM_2.5_ and BC exposures during the third trimester were associated with higher SBP (e.g., 1.0 mmHg; 95% CI: 0.1, 1.8 for a 0.32-μg/m^3^ increase in mean 90-day residential BC). In contrast, O_3_ was negatively associated with SBP (e.g., –2.3 mmHg; 95% CI: –4.4, –0.2 for a 13.5-ppb increase during the 90 days before birth).

**Conclusions:**

Exposures to PM_2.5_ and BC in late pregnancy were positively associated with newborn SBP, whereas O_3_ was negatively associated with SBP. Longitudinal follow-up will enable us to assess the implications of these findings for health during later childhood and adulthood.

**Citation:**

van Rossem L, Rifas-Shiman SL, Melly SJ, Kloog I, Luttmann-Gibson H, Zanobetti A, Coull BA, Schwartz JD, Mittleman MA, Oken E, Gillman MW, Koutrakis P, Gold DR. 2015. Prenatal air pollution exposure and newborn blood pressure. Environ Health Perspect 123:353–359; http://dx.doi.org/10.1289/ehp.1307419

## Introduction

During the prenatal period, a critical period for cardiovascular growth and development, fetuses may be especially vulnerable to adverse health effects of ambient air pollution (Selevan et al. 2000). Earlier studies have reported associations of greater prenatal exposure to air pollution to low birth weight, preterm birth (Bonzini et al. 2010; Bosetti et al. 2010; Ghosh et al. 2007; Glinianaia et al. 2004; Maisonet et al. 2004; Shah and Balkhair 2011; Srám et al. 2005; Stillerman et al. 2008), and higher maternal blood pressure (BP) in pregnancy (van den Hooven et al. 2011). Effects of prenatal air pollution on neonatal BP have not been explored previously, but there is evidence that infant BP is influenced by prenatal maternal conditions including hypertension and maternal drug use (Kent et al. 2009) and by infant’s weight, gestational age, and postnatal age (Gillman et al. 2004; Huxley et al. 2000; Kent et al. 2009; Morrison et al. 2013; Simonetti et al. 2011). In adults, air pollution has been positively associated with BP (reviewed by Brook 2005), although inverse associations between BP and the pollutants particulate matter (PM) and nitrogen oxides (NO_x_) have also been reported (Hampel et al. 2011; Sørensen et al. 2012). Some studies have reported an association between air pollution and higher BP in healthy children (Bilenko et al. 2015; Sughis et al. 2012). Despite the abundant research interest in the early origins of vascular dysfunction, we know of no published study that has assessed the association between prenatal exposure to ambient air pollution and offspring BP.

In this study we investigated associations of prenatal exposures to air pollution [PM ≤ 2.5 μm (PM_2.5_), black carbon (BC), NO_x_, nitrogen dioxide (NO_2_), ozone (O_3_), and carbon monoxide (CO)] with BP in newborns. Although prior studies have generally used PM as a proxy for air pollution exposure (Brook and Rajagopalan 2009), we also considered BC, which is a traffic-related component of PM that has been associated with BP in adults (Mordukhovich et al. 2009). We hypothesized that PM_2.5_, BC, NO_x_, or NO_2_ exposure during pregnancy would be associated with higher newborn BP. We had no prior hypothesis regarding the direction of the association between O_3_ and newborn BP, given the conflicting results reported in adults, including the findings from our research group of lower BP with increased O_3_ among adults with diabetes in Boston (Hoffmann et al. 2012). The latter research shows that the direction of the association may be different in vulnerable subgroups, such as newborns. In addition, although ambient monitors have been used in prior cohort studies to examine effects of pollution exposure, more emphasis is now shifting to spatiotemporal models that capture geographic variations in exposure within a metropolitan area. We have incorporated this for PM_2.5_ and BC.

## Methods

*Study design and participants*. Study subjects were participants in Project Viva, a prospective pre-birth observational cohort study of the influences of antenatal and perinatal factors on maternal and child health. We recruited women who were attending their initial prenatal visit at one of eight urban and suburban obstetrical offices of Harvard Vanguard Medical Associations, a multispecialty group practice located in eastern Massachusetts, between April 1999 and July 2002. Details of recruitment and retention procedures are available elsewhere (Gillman et al. 2004). The human subjects committees of participating institutes approved the study protocols, and all mothers provided written informed consent. All procedures were in accordance with the ethical standards for human experimentation established by the Declaration of Helsinki.

The cohort started with 2,128 live births. We performed in-person visits during the inpatient hospital stay with mothers only on weekdays (*n* = 1,714, 81%), and we measured BP of their newborns (*n* = 1,131, 66%). Reasons for not obtaining a BP measurement were parents not giving consent for measurements (*n* = 328), infant not available when staff was present (*n* = 104), infant transferred to neonatal intensive care unit (*n* = 78), measurements could not be performed (infant too fussy) (*n* = 32), and other reasons (*n* = 41).

## Measurements

*Participant characteristics*. Maternal age (years) was ascertained at enrollment. Maternal third-trimester BP (mmHg) obtained from the medical record was calculated as the average BP between 28 and 32 weeks of gestation. Maternal prepregnancy weight and height [which we used to calculate body mass index (BMI)] and serial urine and BP measurements (which we used to identify gestational hypertension or preeclampsia) were derived from the medical record. Mother’s self-reported race/ethnicity was categorized as black, Hispanic, white, or other. Maternal smoking and physical activity were also self-reported. We obtained birth weight (kilograms) and date of birth from the hospital record. We included socioeconomic status on an individual level estimated by maternal education at enrollment (college graduate vs. less) and at the neighborhood level as median household income derived from the Census 2000 (http://www.census.gov/prod/cen2000/doc/sf3.pdf).

*Outcome*. We measured newborn BP with a Dinamap Pro 100 (Critikon Inc.; or, before 21 February 2001, model 8100) automated oscillometric recorder (GE Medical Services, Tampa, FL) according to a standardized protocol. For each of five measurements taken 1 min apart, we also recorded conditions during the measurement: infant position (in bassinet or held), extremity used (left or right arm), cuff size, and infant state (quiet sleep, active sleep, quiet awake, active awake). We obtained 5 readings on 1,092 infants, 4 readings on 15 infants, 3 readings on 7 infants, 2 readings on 7 infants, and 1 reading on 10 infants, for a total of 5,565 readings on the 1,131 participants. We used systolic BP rather than diastolic BP as our primary outcome because of the validity of its measurement with the oscillometric device and superior prediction of long-term BP (Gillman and Cook 1995).

*Temporally resolved exposure measures*. Ambient concentrations of fine particle mass (PM_2.5_) and BC were measured hourly at a central monitoring site (Harvard Supersite) in Boston. We measured PM_2.5_ concentrations with a tapered element oscillation microbalance (model 1400A; Rupprecht and Pastashnick, East Greenbush, NY), and BC concentrations using an aethalometer (model AE-16; Magee Scientific Co., Berkeley, CA). Air sampling, processing of samples, analysis, and reporting were conducted according to standard operating procedures (Kang et al. 2010). We calculated hourly ambient concentrations of the sum of nitrogen oxides (NO_x_ = NO_2_ + NO), NO_2_, O_3_, and CO by averaging data from the Massachusetts Department of Environmental Protection’s Greater Boston monitoring sites (http://public.dep.state.ma.us/MassAir/) (*n* = 4 for CO and O_3_; *n* = 5 for NO_2_ and NO_x_); thus, these estimates represent a city-wide exposure. Most of the nitrogen oxides emitted by traffic are in the form of nitric oxide (NO); however, part of NO is oxidized to NO_2_ by O_3_. Therefore, we consider NO_x_ as a more robust metric of nitrogen oxide exposures related to traffic, though NO_2_ is more often used as a marker in air pollution studies. Weather data were collected from the National Weather Service Station at Logan Airport (East Boston, MA; http://w1.weather.gov/obhistory/KBOS.html). Missing hourly data for PM_2.5_ and BC (but not other pollution or weather parameters) were imputed. This imputation procedure used long-term trend; seasonality (sine and cosine terms); season (1 = May–September, 0 = otherwise); hour of the day; day of the week; weather (barometric pressure, relative humidity, mean temperature, horizontal visibility, wind direction, and wind speed); gases: CO, NO_2_, SO_2_; ozone during summer months; and interactions with season, wind, and hour of the day. In total, 2% of the 24-hr PM_2.5_ and BC estimates were imputed.

All the pollutants were first summarized in 24-hr (0900–0900 hours) intervals. For calculating the trimester-specific exposures, we calculated gestational age at birth from the last menstrual period or from the second-trimester ultrasound if the two estimates differed by > 10 days (*n* = 200, 9%). Trimester 1 ended at last menstrual period + 93 days, trimester 2 at last menstrual period + 187 days, and trimester 3 at the day before birth. We also calculated different time windows of exposure to air pollution (“moving averages”) from 2 to 7, 14, 30, 60, and 90 days before birth for a more specific evaluation of air pollution exposures close to the time of birth. The moving averages excluded the day of the BP measurement. For each central-site exposure period of interest, for each participant, we required that participants live within 40 km of the Harvard Supersite and that exposure data be available for at least 75% of the time in the specific averaging period; otherwise, the exposure was set to missing.

*Spatially and temporally resolved estimated PM_2.5_ and BC exposures*. Mothers reported their home address at enrollment and updated it at each subsequent study visit, including at birth. We geocoded addresses using ArcGIS (ESRI, Redlands, CA; http://www.arcgis.com) software StreetMap (Firefox, Mozilla) data.

Estimated spatially and temporally resolved PM_2.5_ exposure data were generated by previously described PM_2.5_ prediction models validated for the New England region (Kloog et al. 2012). In brief, we used mixed-effects models with random slopes and intercepts for day to calibrate satellite aerosol optical depth (AOD) data at a resolution of a 10 × 10 km spatial grid (2000–2008) with monitored ground PM_2.5_ measurements. We then used a generalized additive mixed model with spatial smoothing to estimate PM_2.5_ in location–day pairs with missing AOD satellite data (e.g., due to snow, clouds), using regional measured PM_2.5_, AOD values in neighboring cells, and land use variables. “Out-of-sample” 10-fold cross-validation was used to quantify the accuracy of our predictions. That is, 10% of the days were held out of the analysis that generated the prediction, and the accuracy was tested against those held-out measurements. This was repeated 10 times. For days with available AOD data, we found high “out-of-sample” *R*^2^ (mean “out-of-sample” *R*^2^ = 0.87). For days without AOD values, our model performance was also excellent (mean “out-of-sample” *R*^2^ = 0.85). A regression of the held-out data against the predicted had a slope of 1, indicating no bias. To estimate daily spatially and temporally resolved PM_2.5_ exposure, each residence (for the period when the participant lived there) was linked to the 10 × 10 km grid in which it was located. Exposure by trimester and for each averaging period was calculated by averaging daily PM_2.5_ concentrations as described above. Because PM_2.5_ was modeled for 2000–2008, data were missing for newborns born between April and December 1999.

We predicted individual-level estimates of residential BC concentrations from a validated spatiotemporal land-use regression model. Details of the model and its validation have been published earlier (Gryparis et al. 2007; Zanobetti et al. 2014). In short, daily concentrations at the Boston central-site monitor were used as a predictor to reflect average concentration levels for a given day, serving as a direct estimate of the daily time effect. Out-of-sample cross-validation at 32 monitoring sites showed an average correlation of 0.73 between predicted and observed daily BC levels. Data from 148 other stationary air monitors were used to fit the model and estimate the effect of each covariate in the land-use regression model. Covariates in the BC prediction model included measures of land use for each address (cumulative traffic density within 100 m, population density, percent urbanization), location (latitude and longitude), daily meteorological factors (apparent temperature, wind speed, and height of the planetary boundary layer), and temporal factors (day of week and day of season). Separate models were fit for the warm and cold season. Interaction terms between the temporal meteorological predictors and land-use variables allowed for space–time interactions. Regression splines allowed main effect terms to nonlinearly predict exposure levels, and thin-plate splines modeled the residual spatial variability additional spatial variability unaccounted for by the spatial predictors. Daily BC predictions outside of the observed range of the monitored exposure measurements were excluded. To assess the validity of the model, we checked different specifications of the hyperparameters. The results were reasonably robust to even large changes in the specification of the hyperparameters.

*Statistical analyses*. To assess associations between air pollutants and newborn systolic BP, we used mixed-effects models that incorporated each of the up to five BP measurements from each infant as repeated outcome measures (Laird and Ware 1982). Some advantages of this modeling approach, compared with using the average of available measures for each child as the outcome, are that persons with more measurements and less variability among those measurements receive more weight than those with fewer measurements and/or more variability (Fitzmaurice et al. 2011).

We modeled each environmental exposure separately. In all multivariable models, we adjusted for order of measurement and infant state during the measurement. We also adjusted for maternal age, maternal third-trimester BP, and infant’s postnatal age (in hours) and birth weight, because these variables were predictive of newborn BP in an earlier report (Gillman et al. 2004). We adjusted for mother’s race/ethnicity (categorical: black, Hispanic, other, white), and for mother’s educational level (categorical: college degree vs. less), and median neighborhood income (continuous) as indicators of socioeconomic position. Using penalized splines in R version 2.10.0 (R Development Core Team, Vienna, Austria), the model included a variable representing date to take into account seasonality and time trend. Furthermore, we included outdoor temperature (continuous) on the day of the BP measurement. Other weather conditions did not change the estimates of BP (data not shown), and were therefore not included in the model. We visually checked continuous variables for departure from linearity with the outcome with penalized splines, which was not the case for any variable (data not shown). The estimates were scaled to the interquartile range (IQR) (25th to 75th percentile), and reported with their 95% confidence intervals (CIs). Each estimate gives the difference in BP in mmHg for each IQR increase in the pollutant.

We performed a series of sensitivity analyses. First, we considered multi-pollutant models, one with PM_2.5_ and O_3_ together in the same model, and another with O_3_ and NO_x_ together. Second, mother’s BP, infant birth weight, and gestational age could be mediators in the association between trimester-specific exposure to air pollution and newborn BP. Therefore, we also ran a model excluding these potential intermediates. Third, the association of air pollution with BP may differ according to level of specific characteristics, because of differential vulnerability of subgroups to the effect of air pollution on BP. Therefore, using interaction terms, we evaluated infant sex (boy vs. girl), socioeconomic status [both on a neighborhood (median income on a continuous scale) and individual level (college graduate vs. less)], race/ethnicity (black, Hispanic, other vs. white), gestational age (weeks); and for O_3_ season as potential effect modifiers (warm season: May–September; cold season: October–April). We considered effect modification present if the *p*-value of the interaction term was < 0.05. Fourth, for the central monitor exposures we restricted the sample to mothers who lived within 10 km of the Harvard Supersite. Fifth, we restricted the models for the spatiotemporal variables to those children who had information on the temporal variables. Sixth, we repeated the analyses with diastolic BP. Last, we considered additional lifestyle and maternal comorbidities (i.e., maternal physical activity, obesity, parity, preeclampsia, hypertension, and cesarean section) as confounding factors by adding these to the multivariable models and checking the change in estimate for BP.

Statistical analyses were performed using SAS (version 9.2; SAS Institute Inc., Cary, NC, USA) and R version 2.10.0. A *p*-value of < 0.05 was considered statistically significant.

## Results

Table 1 shows the characteristics of the mother–infant pairs in the study, and Supplemental Material, Table S1, shows the association of these characteristics with third-trimester air pollution exposures. The children included in this study were all born at 33–42 weeks, although only 4% were born before 37 weeks of gestation. Mean BP of children born preterm (72.3 ± 9.7 mmHg) and children born term (72.5 ± 8.9 mmHg) was similar. Mean BP varied according to infant’s state (quiet sleep: 70.5 ± 7.9 mmHg; active sleep: 72.1 ± 9.2 mmHg; quiet awake: 74.3 ± 9.3 mmHg; active awake: 77.3 ± 10.3 mmHg). Compared with the 997 mothers of infants who had no BP measurement at birth, the 1,131 participants included in this analysis had higher educational level (67.0% vs. 62.0% completed more than a college degree), had a higher proportion of white race/ethnicity (68.7% vs. 64.0%), and their children had a higher birth weight (3.52 vs. 3.39 kg) and gestational age (39.7 vs. 39.1 weeks). Participants included in the study did not differ substantially with respect to income, smoking status, maternal age, or maternal third-trimester BP compared with those excluded (data not shown) or with the whole study population. A comparison of characteristics between the study population and the analysis sample is presented in Supplemental Material, Table S2. We present the distributions of air pollutant concentrations for the 2-, 30-, and 90-day moving averages in Supplemental Material, Table S3, and the correlation between 2- and 90-day moving averages in Supplemental Material, Tables S4 and S5. Correlations were high for PM_2.5_ but more moderate for BC, especially with increasing number of days averaged. IQR was lower in magnitude with higher number of days averaged.

**Table 1:** Characteristics of 1,131 mother–infant pairs with neonatal blood pressure measurements in Project Viva.

Characteristic	*n* (%) or mean ± SD
Maternal characteristic
Maternal age (years)	32.0 ± 5.3
Maternal education
College graduate	752 (67.0)
Mother’s race/ethnicity
Black	193 (17.2)
Hispanic	70 (6.2)
White	771 (68.7)
Other	89 (7.9)
Median income in neighborhood^*a*^	$58,604 ± $24,833
Maternal smoking during pregnancy
Never	778 (69.2)
Former	213 (19.0)
Smoker	133 (11.8)
Maternal third-trimester SBP (mmHg)^*b*^	111.1 ± 8.1
Gestational age (weeks)	39.7 ± 1.4
Preterm birth (< 37 weeks)	
Yes	47 (4.2)
No	1,084 (95.8)
Child characteristic
Birth weight (kg)	3.52 ± 0.50
Birth weight for gestational age *z*-score	0.20 ± 0.95
Newborn BP (mmHg)	72.5 ± 9.0
Infant state at BP measurement
Quiet sleep	551 (48.7)
Active sleep	131 (11.6)
Quiet awake	351 (31.8)
Active awake	98 (8.7)
^***a***^Census data for address at birth available for 1,125 families. ^***b***^Available for 1,126 mothers.	

In multivariate analyses, newborn SBP was 1.4 mmHg (95% CI: 0.3, 2.5) higher in association with an IQR increase in temporally resolved BC averaged over the third trimester (Table 2). In contrast, SBP was –2.5 mmHg (95% CI: –4.5, –0.4) lower in association with an IQR increase in third-trimester O_3_.

**Table 2:** Association between IQR of trimester-specific estimates of air pollution and SBP (mmHg) in newborns (single-pollutant model).

Exposure	1st Trimester	2nd Trimester	3rd Trimester
Spatiotemporally resolved PM_2.5_
*n*	765	845	970
IQR (μg/m^3^)	2.29	1.97	2.24
β (95% CI)	–0.3 (–1.3, 0.7)	0.4 (–0.4, 1.2)	0.3 (–0.6, 1.2)
Temporally resolved PM_2.5_
*n*	1,032	1,031	1,030
IQR (μg/m^3^)	2.35	1.77	2.05
β (95% CI)	0.1 (–0.9, 1.2)	0.1 (–0.7, 1.0)	0.5 (–0.4, 1.5)
Spatiotemporally resolved BC
*n*	1,099	1,099	1,102
IQR (μg/m^3^)	0.36	0.33	0.33
β (95% CI)	0.00 (–0.8, 0.8)	0.3 (–0.5, 1.0)	1.0 (0.2, 1.8)
Temporally resolved BC
*n*	1,032	1,031	1,030
IQR (μg/m^3^)	0.15	0.16	0.18
β (95% CI)	–0.9 (–1.7, 0.0)	0.3 (–1.1, 1.6)	1.4 (0.3, 2.5)
NO_2_
*n*	1,032	1,031	1,030
IQR (ppm)	2.63	2.96	3.16
β (95% CI)	0.3 (–0.8, 1.5)	–0.5 (–1.9, 0.8)	–0.5 (–1.8, 0.7)
NO_x_
*n*	1,032	1,031	1,030
IQR (ppm)	16.7	17.8	18.2
β (95% CI)	0.3 (–0.8, 1.4)	–1.6 (–2.9, –0.2)	0.6 (–1.2, 2.5)
O_3_
*n*	1,032	1,031	1,030
IQR (ppm)	13.0	12.8	13.6
β (95% CI)	1.2 (–1.0, 3.5)	1.7 (0.3, 3.0)	–2.5 (–4.5, –0.4)
CO
*n*	1,032	1,031	1,030
IQR (ppb)	295.3	269.4	218.1
β (95% CI)	1.9 (–0.1, 3.9)	–2.4 (–3.8, –1.0)	0.1 (–1.5, 1.7)
Estimates are adjusted for neighborhood median income; mother’s age, third-trimester BP, educational level, and race/ethnicity; child birth weight; infant’s age at BP measurement, BP measurement conditions; and time trend.			

Second-trimester temporally resolved PM_2.5_ and BC were not associated with newborn SBP. Higher second-trimester averaged concentrations of the gaseous pollutants NO_x_ and CO were negatively associated with changes in BP. Higher second-trimester averaged concentration of the secondary gaseous pollutant O_3_ was positively associated with changes in SBP.

There were no statistical associations between exposure to any air pollutant in the first trimester and newborn SBP. A key result of this study is our finding of associations of neonatal BP with spatiotemporally resolved as well as temporally resolved air pollution. The association of SBP with spatiotemporally resolved third-trimester BC was consistent with that for temporally resolved BC (1.0 mmHg higher; 95% CI: 0.2, 1.8) for an IQR increase. For PM_2.5_ both temporally and spatiotemporally resolved exposures were not associated with SBP.

**Figure 1:**
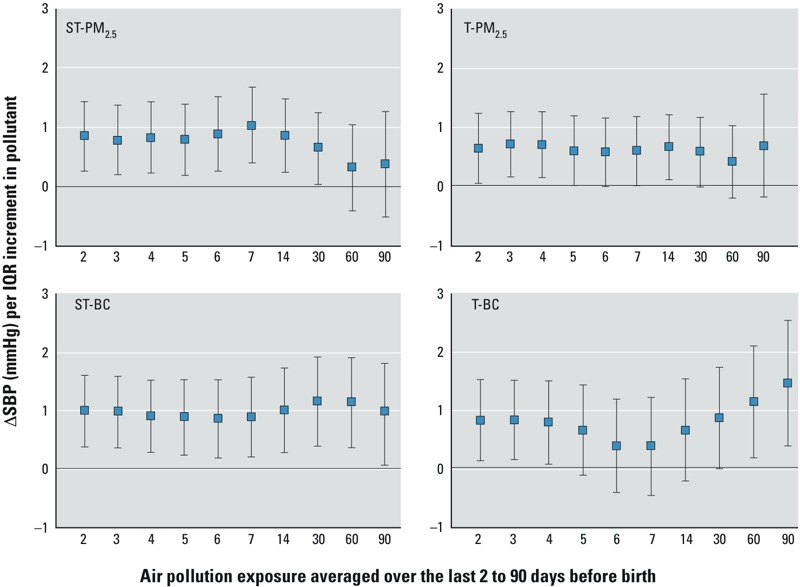
Association of spatiotemporally (ST) resolved PM_2.5_ exposure, temporally (T) resolved PM_2.5_, spatiotemporally resolved BC, and temporally resolved BC during different time windows before birth (“moving averages”) with BP in newborns. Estimates represent mean difference in SBP (95% CI) for an IQR in exposure and are adjusted for neighborhood median income; mother’s age, third-trimester BP, educational level, and race/ethnicity; child birth weight; infant’s age at BP measurement, BP measurement conditions; and time trend.

**Figure 2:**
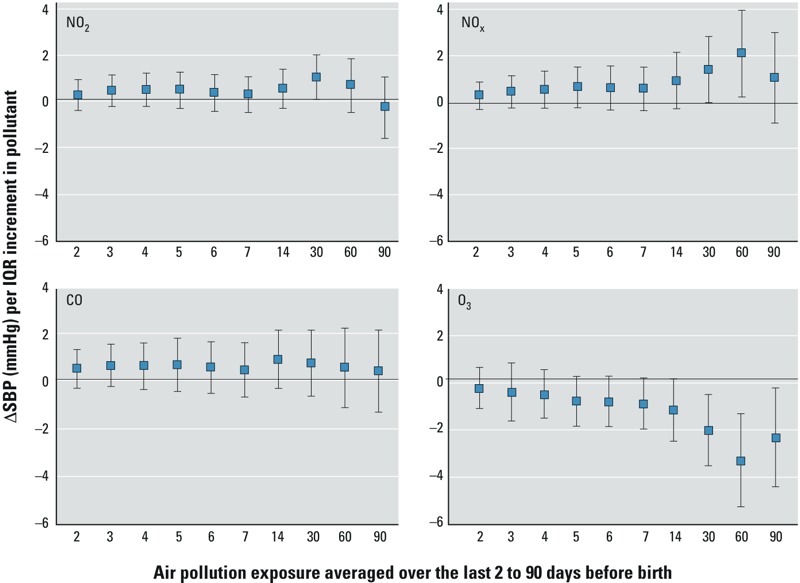
Association of NO_2_, NO_x_, CO, and O_3_ exposure during different time windows before birth (“moving averages”) with BP in newborns. Estimates represent mean difference in SBP (95% CI) for an IQR in exposure and are adjusted for neighborhood median income; mother’s age, third-trimester blood pressure, educational level, and race/ethnicity; child birth weight; infant’s age at BP measurement; and time trend.

For BC and PM_2.5_, cumulative 2- to 90-day exposure was positively associated with SBP, and suggested possible effects of short-term exposures, as well as effects of more long-term exposures (Figure 1). Associations for temporally resolved and spatiotemporally resolved BC and PM_2.5_ were similar, although associations with spatiotemporally resolved PM_2.5_ seemed to decrease for longer-term exposures. For the gases, the predominant associations were related to long-term 30- to 90-day averages (Figure 2), particularly for O_3_ and NO_x_, where O_3_ was negatively associated with SBP and NO_x_ was positively associated with SBP.

We performed a series of sensitivity analyses. First, we considered two-pollutant models for 60-day moving averages that included temporally resolved BC and O_3_ or NO_x_ and O_3_. The correlation between the 60-day moving averages of BC and O_3_ was –0.17. In the multi-pollutant model, the associations for BC and O_3_ with newborn SBP were attenuated when both were included in the model: BC from 1.2 mmHg (95% CI: 0.2, 2.1) to 0.7 mmHg (95% CI: –0.3, 1.6), and estimates for O_3_ attenuated from –3.3 mmHg (95% CI: –5.3, –1.3) to –2.8 mmHg (95% CI: –4.9, –0.8). The correlation between the 60-day moving averages of NO_x_ and O_3_ was –0.9. In the multi-pollutant model the association for NO_x_ with newborn SBP was attenuated when including both NO_x_ and O_3_ in the model: NO_x_ changed from 2.1 mmHg (95% CI: 0.2, 3.9) to –0.7 mmHg (95% CI: –3.4, 2.1), but the estimate for O_3_ increased from –3.3 mmHg (95% CI: –5.3, –1.3) to –3.9 mmHg (95% CI: –5.8, –1.9). In a two-pollutant model with PM_2.5_ and O_3_, the estimates were fairly similar to the estimates of the one-pollutant model (data not shown). To assess the robustness of the short-term averages, we also ran a two-pollutant model including short-term BC/PM_2.5_ and O_3_; estimates of BC, PM_2.5_, and O_3_ were fairly similar to the estimates of the one-pollutant model (data not shown).

Excluding mother’s BP and birth weight from the model did not materially change the estimated associations, suggesting that these factors did not mediate the relationship (data not shown).

Effect modification models resulted in interaction terms for child’s sex, socioeconomic status, race/ethnicity, gestational age/preterm birth, and season (tested for O_3_) that were mostly associated with *p*-values > 0.10 (data not shown), so we did not stratify the results. Restricting the sample to mothers who lived within 10 km (2,776/5,565 observations) of the monitoring site produced similar estimates, and restricting the spatiotemporal models to participants who also had data for the temporal models (5,140/5,565) did not change the estimates (data not shown).

Last, repeated analyses with diastolic BP were similar in relative magnitude and direction, but seemed weaker overall (see Supplemental Material, Table S6 and Figures S1 and S2).

In general, adjusting for maternal physical activity, obesity, parity, preeclampsia, hypertension, and cesarean section did not substantially influence associations between newborn SBP and air pollutants (data not shown).

## Discussion

In this study of prenatal air pollution and newborn BP, we found that higher short-term and longer-term trimester-specific concentrations of PM_2.5_ and BC were associated with higher newborn BP, whereas higher concentrations of NO_2_, NO_x_, and O_3_ were associated with lower and higher newborn BP (depending on the pregnancy period). Associations were mainly seen in the second and third trimesters, and for PM_2.5_ and BC for both shorter- and, particularly for BC, longer-term cumulative exposure. For gaseous pollution, BP changes were associated with longer-term exposures.

BP is a function of cardiac output and peripheral vascular resistance, each of which can be influenced by a number of factors. Epidemiological and experimental studies in adults suggest that underlying mechanisms for short-term air pollution effects on increased BP relate to autonomic imbalance and systemic oxidative/inflammatory responses promoting vascular endothelial dysfunction (Brook 2005). A growing literature supported by animal models suggests that longer-term exposures to particle pollution in adults can lead to atherosclerosis and vascular remodeling (Pope et al. 2004; Soares et al. 2009; Sun et al. 2005). These short- and long-term vascular responses could be applicable to the fetus as well: At least one study has found adverse effects of antenatal air pollution (PM_2.5_, CO, NO_2_, sulfur dioxide) on placental vascular structure in a rodent model (Veras et al. 2008). Adjustment for the potential mediators such as maternal BP or birth weight did not alter associations, suggesting that air pollution may have influenced cardiac output and structure directly through increasing placental vascular or fetal vascular resistance and cardiac output. However, experimental studies in animals of air pollution in pregnancy and BP in offspring are lacking, and human interventions investigating this issue may be difficult to perform.

In an earlier study, we reported opposing associations of particle pollutants and O_3_ with BP in an adult population with diabetes after adjustment for season and ambient temperature (Hoffmann et al. 2012). We found that exposure to particle pollutants was associated with higher BP, whereas exposure to O_3_ was associated with lower BP in the third trimester, but with higher BP in the second trimester. In addition, exposure to CO and NO_x_ in the second trimester was associated with lower BP. An earlier study reported an inverse association between nitrogen oxides and BP in adults (Sørensen et al. 2012). Thus, particle pollutants and gaseous pollutants may have different mechanisms underlying the air pollution–newborn BP association. A fall in cardiac output has been shown in mice exposed to O_3_ (Lee and Pisarri 2001), but how this phenomenon might work *in utero* is unknown. Alternatively, O_3_ was strongly inversely correlated with NO_2_ (*r*_90 days average_ = –0.69) and NO_x_ (*r*_90 days average_ = –0.92) in our study, which is consistent with its chemical properties: NO_x_ is a primary vehicular pollutant that quenches O_3_. In addition, ground-level O_3_ is higher with higher temperature and low wind speed. Although the association of O_3_ with lower BP may be spurious due to its negative correlation with NO_x_ and other pollutants (Brook et al. 2009), the strong negative correlations make it difficult to disentangle effects. In two-pollutant models, O_3_ was robust to adjustment for BC and NO_2_, but the estimates for BC and NO_2_ were attenuated.

The consistent findings of short-term PM associations, and short- and longer-term (30- to 90-day and trimester-specific) BC associations with BP suggest that these associations were not confounded by season or other unmeasured exposures that season may represent. With the stationary site–measured gases, we find long-term associations only with BP. Because of this, despite our adjustment with penalized splines for season and for year-by-season trends, we cannot rule out the possibility that whether the trimester-specific or 30- to 90-day average gas (NO_x_ or O_3_) associations with BP may be partly confounded by unmeasured factors related to these 30- to 90-day periods. This is, of course, a consideration in all studies that use trimester-specific or 90-day averaged temporally but not spatially resolved pollution, because time periods shorter than 365 days overlap with season. Future modeling of spatiotemporally resolved NO_x_ in our study will help us further disentangle season from pollution associations.

This study has many strengths including careful measures of newborn BP and information on a number of antenatal predictors and potential confounders. Nevertheless, some limitations exist. The precision of exposure estimates may have been reduced by using central-site measures for many exposures of interest. However, this was not reflected in the estimates because the third-trimester BC associations with BP were less strong for the spatiotemporal estimates of BC than for the temporal measures of BC. It is possible that regional BC may better reflect exposure than residence-specific BC, because participants may not be at home during the day. In contrast with BC, third-trimester spatiotemporal PM_2.5_ estimates had larger, more precise associations with BP than did central-site measures of PM, even though the satellite-based estimates of PM_2.5_ were based on a 10-km, not a residence-specific, spatial resolution. A limitation of the present study is the spatial resolution of 10 × 10 km and the missing data for the spatiotemporal estimates of PM_2.5_. However, the missing data are not likely to be associated with the exposure and/or outcome variable and therefore probably only reduce precision. Although we have adjusted for season, some seasonal components may still be mixed with the effects of the 90-day and trimester-specific estimates of air pollutant. Included and excluded participants differed according to maternal race and education. This could have led to selection bias if the association between air pollution and BP differed in those who were not in the study. Although this is unlikely, we cannot completely rule out the possibility. Finally, our study consisted of healthy neonates, and therefore the results cannot be generalized to less healthy infants, including children born preterm.

Circulatory changes in the first hours and days of life are profound; some of the cardiovascular measures at birth may represent transient responses and others may have long-term implications. Neonatal BP may have a different meaning than BP measured later in infancy, childhood, or adulthood. From infancy onward, children tend to maintain their BP ranking, meaning that infants with relatively high BP are more likely to have high BP in adulthood (Chen and Wang 2008). The literature on the implication of neonatal BP for later cardiovascular disease or risk of hypertension is sparse (de Swiet et al. 1980; Higgins et al. 1980; Levine et al. 1978; Schachter et al. 1979; Zinner et al. 1985). To our knowledge there is no follow-up study that assessed the association between neonatal BP and later cardiovascular health.

## Conclusion

Our study contributes unique insight into prenatal pollution exposures and neonatal BP. In summary, we found that third-trimester antenatal exposures to BC and, to a lesser extent, PM_2.5_ were associated with higher newborn BP, whereas concentrations of O_3_ in the third trimester were associated with lower BP. Future follow-up will indicate whether these associations persist into later childhood.

## Supplemental Material

(420 KB) PDF
