# Supplementary material for: Prenatal Air Pollution Exposure and Newborn Blood Pressure
Source: Environ Health Perspect. 2015 Jan 27;123(4):353–9. doi: 10.1289/ehp.1307419 (PMC4384198; doi:10.1289/ehp.1307419)
Supplement: (420 KB) PDF [file ehp.1307419.s001.508.pdf]

Environmental Health Perspectives doi:10.1289/ehp.1307419

Supplemental Material

Prenatal Air Pollution Exposure and Newborn Blood Pressure

Lenie van Rossem, Sheryl L. Rifas-Shiman, Steven J. Melly, Itai Kloog, Heike Luttmann-Gibson, Antonella Zanobetti, Brent A. Coull, Joel D. Schwartz, Murray A. Mittleman, Emily Oken, Matthew W. Gillman, Petros Koutrakis, and Diane R. Gold

Table S1. Bivariate associations between covariates and interquartile range of third trimester air pollution exposures in the study.

Variable	$PM_{2.5}$ (µg/m^3) Spatiotemporally resolved	$PM_{2.5}$ (µg/m^3) Temporally resolved	BC (µg/m^3) Spatiotemporally resolved	BC (µg/m^3) Temporally resolved	NO_X (ppb)	NO_2 (ppb)	O_3 (ppb)	CO (ppb)
Interquartile range	2.24	2.05	0.33	0.18	3.16	18.2	13.6	218.1
**Maternal characteristics**								
Maternal age (per 5 years)	0.01 (-0.03, 0.05)	0.04 (-0.01, 0.08)	-0.09 (-0.13, -0.05)	0.02 (-0.01, 0.06)	-0.03 (-0.06, 0.00)	-0.04 (-0.07, -0.00)*	0.02 (-0.01, 0.05)	-0.05 (-0.09, -0.01)*
Maternal education College graduate	0.02 (-0.08, 0.11)	-0.02 (-0.12, 0.08)	0.21 (0.12, 0.30)*	0.06 (-0.02, 0.14)	0.002 (-0.07, 0.07)	-0.03 (-0.10, 0.05)	-0.01 (-0.08, 0.05)	0.01 (-0.09, 0. 10)
Mother's race/ethnicity								
Black	0.01 (-0.10, 0.13)	-0.06 (-0.18, 0.07)	0.38 (0.26, 0.49)*	0.04 (-0.06, 0.14)	-0.01 (-0.10, 0.08)	-0.03 (-0.13, 0.07)	0.00 (-0.08, 0.09)	0.00 (-0.12, 0.12)
Hispanic	0.07 (-0.10, 0.25)	0.11 (-0.08, 0.29)	0.48 (0.31, 0.65)*	-0.03 (-0.19, 0.11)	-0.05 (-0.19, 0.09)	-0.02 (-0.17, 0.13)	0.05 (-0.08, 0.17)	-0.12 (-0.30, 0.07)
Other	0.16 (-0.00, 0.32)	-0.04 (-0.21, 0.13)	0.35 (0.20, 0.51)*	0.00 (-0.13, 0.14)	0.17 (0.04, 0.29)*	0.14 (0.01, 0.28)*	-0.14 (-0.26, -0.03)	0.17 (0.00, 0.34)*
White	ref	ref	ref	ref	ref	ref	ref	ref
Median income in neighborhood	0.00 (0.00, 0.00)	0.00 (0.00, 0.00)	0.00 (0.00, 0.00)	0.00 (0.00, 0.00)	0.00 (0.00, 0.00)	0.00 (0.00, 0.00)	0.00 (0.00, 0.00)	0.00 (0.00, 0.00)
Maternal smoking during pregnancy								
Never	ref	ref	ref	ref	ref	ref	ref	ref
Former	-0.06 (-0.17, 0.05)	-0.03 (-0.15, 0.09)	-0.05 (-0.16, 0.06)	0.02 (-0.08, 0.12)	0.01 (-0.07, 0.10)	0.00 (-0.09, 0.10)	-0.02 (-0.11, 0.06)	0.05 (-0.07, 0.17)
Smoker	-0.07 (-0.17, 0.05)	-0.04 (-0.19, 0.10)	0.08 (-0.06, 0.21)	-0.08 (-0.20, 0.04)	-0.01 (-0.12, 0.09)	0.02 (-0.09, 0.14)	0.04 (-0.06, 0.14)	-0.02 (-0.16, 0.12)
Maternal third trimester blood pressure (mmHg)	-0.01 (-0.01, -0.00)*	-0.01 (-0.01, -0.00)*	-0.00 (-0.01, 0.00)*	-0.01 (-001, -0.00)*	0.00 (-0.00, 0.01)	0.00 (-0.00, 0.01)	-0.00 (-0.00, 0.00)	0.00 (0.00, 0.00)
Gestational age (weeks)	0.00 (-0.03, 0.03)	0.01 (-0.02, 0.05)	-0.01 (-0.04, 0.03)	0.00 (-0.02, 0.03)	-0.01 (-0.04, 0.01)	-0.02 (-0.04, 0.01)	0.01 (-0.02, 0.03)	-0.05 (-0.14, 0.04)
**Child characteristics**								
Birth weight (kg)	-0.03 (-0.12, 0.06)	0.05 (-0.04, 0.14)	-0.13 (-0.21, -0.04)*	-0.01 (-0.09, 0.06)	-0.05 (-0.12, 0.02)	-0.04 (-0.12, 0.03)	0.05 (-0.02, 0.11)	-0.05 (-0.14, 0.04)

Number represent b (95% CI).

PM: Particulate Matter; BC: Black Carbon; *P<0.05.

Table S2. Comparison of characteristics between study population and population for analyses.

Characteristics	Total sample (n=2,128)<br>N (%) or Mean ± SD	Population for analyses (n=1,131)<br>N (%) or Mean ± SD
**Maternal characteristics**		
Maternal age	31.8 ± 5.2	32.0 ± 5.3
Maternal education = College graduate	1360 (64.6)	752 (67.0)
Mother's race/ethnicity		
Black	348 (16.5)	193 (17.2)
Hispanic	154 (7.3)	70 (6.2)
White	1399 (66.5	771 (68.7)
Other	203 (9.7)	89 (7.9)
Median income in neighborhood[a]	58,585 ± 24,517	58,604 ± 24,833
Maternal smoking during pregnancy		
Never	1443 (68.5)	778 (69.2)
Former	398 (18.9)	213 (19.0)
Smoker	266 (12.6)	133 (11.8)
Maternal third trimester systolic blood pressure (mmHg)[b]	111.2 ± 8.4	111.1 ± 8.1
Gestational age (weeks)	39.4 ± 2.0	39.7 ± 1.4
Preterm birth (<37 weeks)	154 (7.2)	47 (4.2)
**Child characteristics**		
Birth weight (kg)	3.46 ± 0.59	3.52 ± 0.50
Birth weight for gestational age z -score	0.17 ± 0.97	0.20 ± 0.95

Table S3. Descriptive statistics of air pollutants among 1,131 mother-infant pairs with neonatal blood pressure measurements in Project Viva.

Exposure	2 days 25th percentile	2 days Median	2 days 75th percentile	30 days 25th percentile	30 days Median	30 days 75th percentile	90 days 25th percentile	90 days Median	90 days 75th percentile
Spatiotemporally resolved $PM_{2.5}$ ($\mu g/m^3$)[a]	7.9	10.5	14.6	10.2	11.5	12.9	10.7	11.8	13.0
Temporally resolved $PM_{2.5}$ ($\mu g/m^3$)[b]	7.4	10.3	14.0	9.7	10.7	12.2	10.1	10.9	12.1
Spatiotemporally resolved BC ($\mu g/m^3$)[c]	0.43	0.62	0.89	0.50	0.67	0.85	0.53	0.68	0.85
Temporally resolved BC ($\mu g/m^3$)[b]	0.58	0.77	1.07	0.73	0.84	0.94	0.74	0.86	0.93
NO_2 (ppb)[b]	18.2	20.9	24.7	19.9	21.5	23.2	20.3	21.6	23.4
NO_x (ppb)[b]	29.9	38.0	50.4	33.6	40.0	53.6	35.0	43.4	53.2
O_3 (ppb)[b]	15.5	22.6	30.4	16.1	24.1	30.0	15.7	23.4	29.2
CO (ppm)[b]	0.33	0.43	0.62	0.37	0.45	0.60	0.39	0.49	0.62

[a]PM = Particulate Matter; available for 1048 (2 days), 1031 (30 days), and 990 (90 days) mother-infant pairs. [b]Available for 1045 (2 days and 30 days), and 1043 (90 days) mother-infant pairs. [c]BC = Black carbon; available for 1117 (2 days and 30 days), and 1116 (90 days) mother-infant pairs.

Table S4. Correlations between air pollutants for 2 day moving averages.

Exposure	$PM_{2.5}$ (spatiotemporal)	$PM_{2.5}$ (temporal)	BC (spatiotemporal)	BC (temporal)	NO_2	NO_x	O_3	CO
Spatiotemporally resolved $PM_{2.5}$	1	0.88	0.50	0.57	0.41	0.29	0.20	0.29
Temporally resolved $PM_{2.5}$		1	0.48	0.61	0.41	0.25	0.27	0.21
Spatiotemporally resolved BC			1	0.74	0.49	0.58	-0.27	0.45
Temporally resolved BC				1	0.47	0.50	-0.16	0.37
NO_2					1	0.80	-0.26	0.65
NO_x						1	-0.60	0.83
O_3							1	-0.50
CO								1

Table S5. Correlations between air pollutants for 90 day moving averages.

Exposure	$PM_{2.5}$ (spatiotemporal)	$PM_{2.5}$ (temporal)	BC (spatiotemporal)	BC (temporal)	NO_2	NO_x	O_3	CO
Spatiotemporally resolved $PM_{2.5}$	1	0.79	0.40	0.47	0.07	0.13	-0.13	-0.05
Temporally resolved $PM_{2.5}$		1	0.10	0.38	-0.30	-0.30	0.26	-0.48
Spatiotemporally resolved BC			1	0.40	0.06	0.24	-0.35	0.15
Temporally resolved BC				1	-0.41	-0.08	-0.18	-0.18
NO_2					1	0.88	-0.69	0.74
NO_x						1	-0.92	0.84
O_3							1	-0.70
CO								1

Table S6. Association between interquartile range of trimester specific estimates of air pollution and diastolic blood pressure (mmHg) in newborns (single-pollutant model).

**Exposure**	**1st Trimester**	**2nd Trimester**	**3rd Trimester**
**Spatiotemporally resolved $PM_{2.5}$[a]**			
N	765	845	970
β (95% CI)	0.15 (-0.56, 0.86)	0.23 (-0.36, 0.82)	0.17 (-0.46, 0.80)
**Temporally resolved $PM_{2.5}$**			
N	1,032	1,031	1,030
β (95% CI)	-0.39 (-1.13, 0.35)	0.15 (-0.46, 0.76)	0.21 (-0.42, 0.84)
**Spatiotemporally resolved BC[b]**			
N	1,099	1,099	1,102
β (95% CI)	0.19 (-0.40, 0.78)	0.39 (-0.16, 0.94)	**0.82 (0.25, 1.38)**
**Temporally resolved BC**			
N	1,032	1,031	1,030
β (95% CI)	-0.53 (-1.14, 0.08)	0.38 (-0.56, 1.32)	0.32 (-0.42, 1.06)
**NO_2**			
N	1,032	1,031	1,030
β (95% CI)	-0.02 (-0.78, 0.74)	-0.05 (-0.98, 0.83)	-0.06 (-0.92, 0.81)
**NO_X**			
N	1,032	1,031	1,030
β (95% CI)	0.31 (-0.87, 1.49)	-0.56 (-1.44, 0.32)	0.47 (-0.76, 1.70)
**O_3**			
N	1,032	1,031	1,030
β (95% CI)	0.13 (-1.40, 1.66)	0.48 (-0.40, 1.36)	-0.94 (-2.39, 0.51)
**CO**			
N	1,032	1,031	1,030
β (95% CI)	0.91 (-0.48, 2.30)	-0.92 (-1.90, 0.06)	0.35 (-0.71, 1.41)

Estimates are adjusted for neighborhood median income; mother's age, third trimester blood pressure, educational level, and race/ethnicity; child birth weight; infant's age at BP measurement, BP measurement conditions; and time trend.

[a]PM = Particulate matter; [b]BC = Black Carbon.

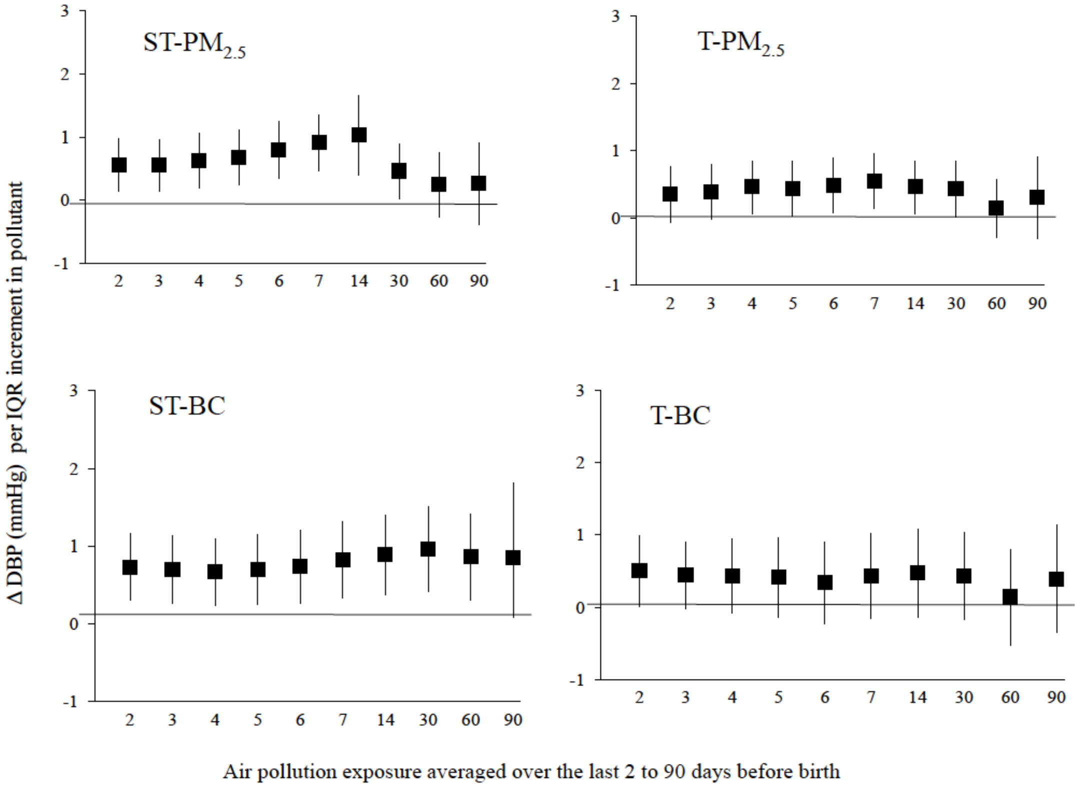

Figure S1. Association of spatiotemporally resolved $PM_{2.5}$ exposure; temporally resolved $PM_{2.5}$; spatiotemporally resolved BC; and temporally resolved BC during different time windows before birth ("moving averages") with diastolic blood pressure in newborns. Estimates represent mean difference in systolic blood pressure (95% confidence interval) for an IQR in exposure and are adjusted for neighborhood median income; mother's age, third trimester blood pressure, educational level, and race/ethnicity; child birth weight; infant's age at BP measurement, BP measurement conditions; and time trend.

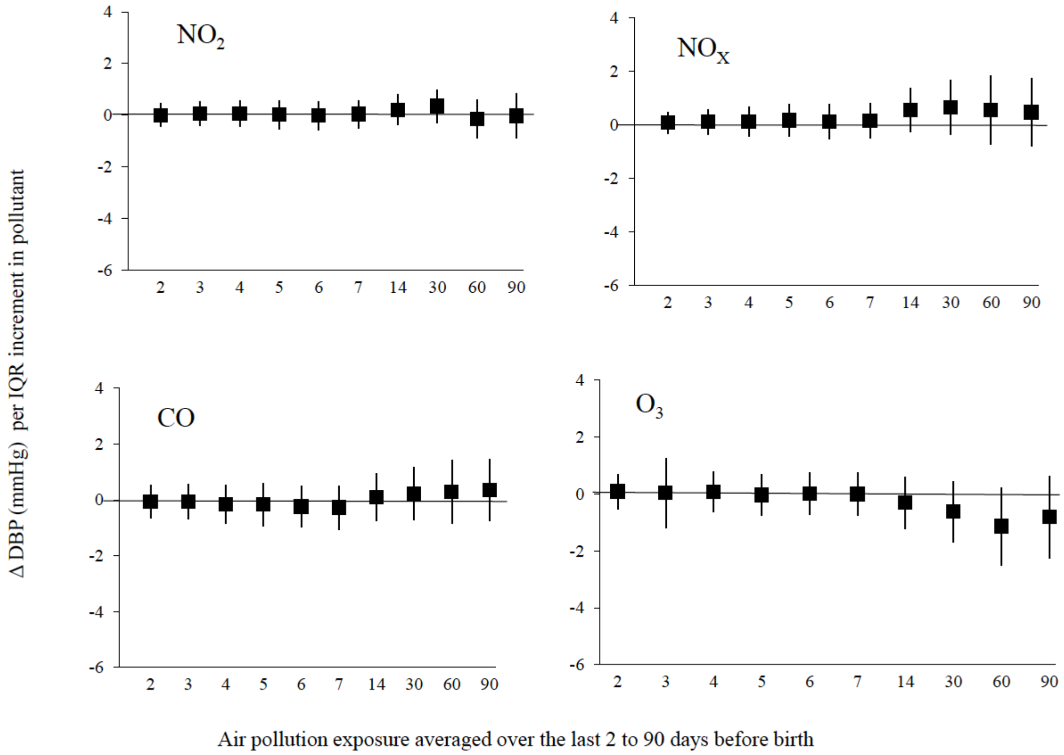

Figure S2. Association of NO_2; NO_x; CO; and O_3 exposure during different time windows before birth ("moving averages") with diastolic blood pressure in newborns. Estimates represent mean difference in systolic blood pressure (95% confidence interval) for an IQR in exposure and are adjusted for neighborhood median income; mother's age, third trimester blood pressure, educational level, and race/ethnicity; child birth weight; BP measurement conditions; and seasonality.
